# Mechanisms of Cbl-Mediated Ubiquitination of Proteins in T and Natural Killer Cells and Effects on Immune Cell Functions

**DOI:** 10.3390/life14121592

**Published:** 2024-12-03

**Authors:** Pulak Ranjan Nath, Noah Isakov

**Affiliations:** 1 Lentigen Technology Inc., A Miltenyi Biotec Company, 910 Clopper Road, Gaithersburg, MD 20878, USA; hellopran2000@gmail.com; 2 The Shraga Segal Department of Microbiology, Immunology and Genetics, Faculty of Health Sciences, Ben Gurion University of the Negev, P.O. Box 653, Beer Sheva 84105, Israel

**Keywords:** Cbl, ubiquitin, signal transduction, proteasomal degradation, T cell, NK cell, CAR-T cells, cancer immunotherapy, TCR, C3G, LAT

## Abstract

Post-translational ubiquitination is an essential mechanism for the regulation of protein stability and function, which contributes to the regulation of the immune system. Cbl, an E3 ubiquitin ligase, is particularly well-characterized in the context of T and NK cell signaling, where it serves as a key regulator of receptor downstream signaling events and as a modulator of cell activation. Cbl promotes the proteasomal degradation of TCR/CD3 subunits as well as the protein kinases Fyn and Lck in T cells. Additionally, the scaffold protein linker for activation of T cells (LAT) is a universal target for Cbl-mediated ubiquitination and degradation in both T and NK cells. Recent findings suggest that CrkII-mediated ubiquitination and degradation of C3G by Cbl during early T cell activation may also be relevant to NK cell signaling. Given its role in modulating immune responses and its manageable impact on autoimmunity, Cbl is being investigated as a target for cancer immunotherapy. This review explores the ubiquitin ligase activity of Cbl and its implications for CAR T and NK cell immunotherapies.

## 1. Introduction

Post-translational modifications of proteins regulate their structure and function and turn on or off signal transduction mechanisms during cell stimulation [1,2]. Ubiquitination is one such post-translational modification that controls multi-faceted cellular processes, including proteasomal degradation of proteins, endocytosis of receptors, protein–protein interactions, kinase activation, gene transcription, and assembly of signaling complexes [3]. Ubiquitination therefore controls the function, localization, and stability of proteins [4] and the extent and intensity of different types of immune responses [5,6]. In the event of ubiquitination, a short chain of ubiquitin (~76 amino acids) is covalently attached to different internal lysine (K) residues or the N-terminal methionine (M1) residue within a substrate protein [7]. It is a stepwise process, starting with the activity of E1 ubiquitin-activating enzymes, followed by the activity of E3 ubiquitin-ligating enzymes [8]. The substrate protein may bind to one ubiquitin (monoubiquitination), multiple single ubiquitins (multi-monoubiquitination), or polyubiquitin chains (polyubiquitination). Monoubiquitination and multi-monoubiquitination mediate the endocytosis of membrane receptors, which may lead to protein re-localization, degradation, and association with other molecules [9,10]. While E3 ligases promote protein ubiquitination, proteases called deubiquitinases (DUBs) counteract the process by cleaving the ubiquitin chains [3]. A large number of mammalian E3 ligases (~600) and DUBs (~100) orchestrate the tightly controlled and substrate-specific processes of ubiquitination and deubiquitination that regulate specific cellular functions [3,7].

## 2. Cbl, an E3 Ubiquitin Ligase

**Figure 1:**
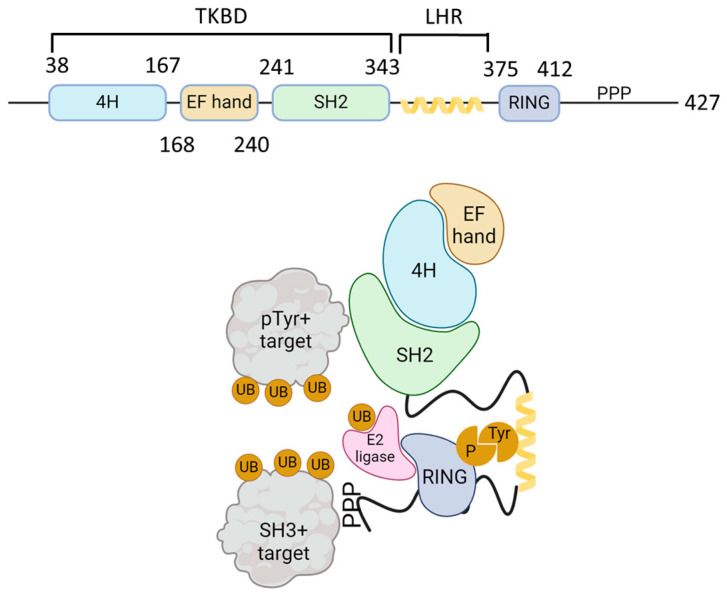
Schematic structure of Cbl-b and mechanism of action. The upper figure shows the schematic structure of Cbl-b protein. The N-terminal tyrosine kinase binding domain (TKBD) is composed of a four-helical bundle, a calcium-binding EF-hand, and an SH2 domain. A short helical region links the N-terminal TKBD and C-terminal RING finger domain, followed by a polyproline-rich region (PPP). The lower figure models the Cbl ubiquitin ligase function. SH2 domain (green) and PPP-regions of Cbl-b bind to phospho-tyrosine (pTyr) and SH3-domain-bearing proteins, respectively. Ubiquitin (UB, orange) is activated by E1 ligase and transferred to a ubiquitin-conjugating enzyme (E2 ligase, pink). E2 ligase interacts with Cbl-b via its RING domain (blue) and brings UB to the proximity of Cbl-b-interacting proteins and promotes their mono- or polyubiquitination. The figure was created using BioRender.com (www.biorender.com, accessed on 24 November 2024).

Casitas B-lineage lymphoma (Cbl) is an adaptor molecule that also functions as an E3 ubiquitin ligase. In mammals, the Cbl family of proteins contains three distinct members: c-Cbl, Cbl-b, and Cbl-3 [11]. These three members share a conserved N-terminus tyrosine kinase binding domain (TKBD), in addition to an alpha-helical linker region (LHR) and a catalytic RING (really interesting group) finger domain (Figure 1). The TKBD is composed of three subdomains: a 4-helix bundle (4H), a calcium-binding domain with an EF-hand fold (EF hand), and a Src homology region 2 (SH2) domain variant, which together make up the phospho-tyrosine (pTyr) recognition module of Cbl-b. The LHR contains an N-terminal linker loop, a helix, and a C-terminal linker loop and contains a conserved tyrosine 363 residue that is essential for the E3 ligase activity. Phosphorylation of Tyr363 activates Cbl-b from its autoinhibited state. The RING finger domain directly associates with ubiquitin (UB)-conjugated E2 proteins. A less conserved C-terminal region that consists of proline-rich (PPP) regions, tyrosine phosphorylation sites, and a ubiquitin-associated (UBA)/leucine zipper (LZ) domain is found in c-Cbl and Cbl-b [12]. PPP regions bind to SH3 domains of target signaling proteins, and tyrosine residues that become phosphorylated interact with key SH2 domain-containing target signaling proteins. The E1 ubiquitin-activating enzyme activates UB and transfers it to a ubiquitin-conjugating enzyme (E2), which interacts directly with the Cbl-b ubiquitin ligase (E3) and transfers UB to the target proteins (Figure 1).

Cbl-b is the best characterized ubiquitin ligase involved in T and NK cell signaling events. Cbl-b functions as a key modulator of T cell activation and downregulates T cell functions in the absence of CD28 costimulation [13]. It is recruited to the TCR-CD3 complex upon cell activation [14] and accumulates at the immunological synapse [14,15]. It functions as a scaffold for signal transducing effector molecules, including lymphocyte cell-specific protein-tyrosine kinase (Lck), SH2-domain-containing leukocyte protein of 76 kDa (SLP76), zeta chain-associated protein 70 (ZAP70), Vav1, protein kinase C theta (PKCθ), and phosphoinositide-3 kinase (PI3K) [16]. By interacting via its RING and TKB domains with E2 ubiquitin-conjugated enzymes and substrate proteins, respectively, Cbl-b catalyzes the transfer of ubiquitin from E2 to lysine residues on the substrate proteins [17].

Ubiquitination of target proteins by Cbl-b may lead to their proteasomal degradation, as was demonstrated for the TCR/CD3 subunits [18,19], as well as the Fyn [20] and Lck protein tyrosine kinases [21]. Apart from promoting proteasomal degradation, Cbl-b enables the Lys33-linked polyubiquitylation of the CD3ζ chain, thereby preventing the CD3ζ phosphorylation and association with the downstream protein tyrosine kinase, ZAP70 [22]. Cbl-b-mediated ubiquitination of phospholipase C gamma 1 (PLCγ1) and the PI3K regulatory subunit, p85, downregulates activation signals in T cells [23]. As a result, T cells from Cbl-b-deficient mice showed hyperactive phenotypes [24] and augmented anti-tumor activity in vivo [13,25,26,27,28]. Cbl-b also directly ubiquitinates SMAD7, thereby promoting transforming growth factor beta (TGF-β) signaling, which contributes to the overall immunosuppressive phenotype [29]. In agreement, mice with *Cbl-b*-deficient T cells not only consistently and spontaneously reject diverse types of tumors but also confer anti-tumor immunity upon their adoptive transfer to tumor-bearing mice. Furthermore, these T cells display long-term memory for the tumor antigen [25,30], while deficiency of Cbl-b limits T cell exhaustion in the tumor environment. CRISPR/Cas9-mediated depletion of Cbl-b restores the expression of inflammatory cytokines and cytotoxic molecules in exhausted programmed cell death 1 (PD-1)+T cell immunoglobulin mucin 3 (Tim3)+ T cells [31]. T cells from *Cbl-b*-deficient mice are not anergized upon their in vitro exposure to ionomycin or in vivo in P14 or OT-II TCR transgenic mice [32,33]. They display resistance to Treg and TGF-β-induced suppression [13,26,34], as well as to inhibition by the immune checkpoint agonist, PD-L1 [34,35,36]. There were concerns that Cbl-b depletion might increase the frequency and/or intensity of autoimmune diseases, as was demonstrated for experimental models of encephalomyelitis [24], autoimmune arthritis [33], and type 1 diabetes [37]. In most cases, the Cbl-b-deficient mice only develop a mild and non-lethal autoimmune phenotype [25,28]. Importantly, no signs of severe autoimmune toxicity were observed in the Cbl-b-deficient mice even a year following their rejection of the tumor cells [13,25,26,28,38]. Similarly, even though tumor antigens were expressed in distal organs [27], no off-target toxicity or autoimmune injury were reported in wild-type mice reconstituted with *Cbl-b*-knockout or -knockdown CD8+ T-cells [13,25,27,30,39,40]. Thus, due to its inhibitory effects on multiple checkpoints in immunocytes and because of the relatively tolerable effects of Cbl-b deficiency on autoimmune-related hazards, Cbl-b has become an attractive cancer immunotherapy target and is being exploited in a large number of preclinical studies and clinical trials [27,41] (Supplementary Table S1, https://clinicaltrials.gov/search?term=Cbl, accessed on 14 July 2024).

## 3. Effect of Cbl on T Cells

Cbl-b limits the overall differentiation of T cell subsets; in Cbl-b-deficient mice, an increase in the differentiation of Th2, Th9, and Th17 cells has been observed [42]. Mechanistically, this effect has been attributed to the increase in the Janus kinase (JAK)/signal transducer and activator of transcription 6 (Stat6) signaling [42]. Cbl-b is also reported to promote peripheral Treg development, converting CD4+ CD25- cells into CD4+ CD25+ FoxP3+ T cells via the Akt2 signaling pathway [43]. Tregs are integral to the regulation of autoimmune and anti-tumor immune responses. A recent study reported that the absence of Cbl-b rendered T cells impartial to Treg suppression, leading to improved anti-tumor immunity despite Tregs being present in the tumor. Cbl-b KO CD4+FoxP3- T cells hyperproduced IL-2 and upregulated IL-2Rα, which essentially helped CD4+ FoxP3- T cells to escape suppression by Tregs [44].

Cbl-b-deficient CD8+ T cells are also resistant to Treg or TGF-β-mediated suppression [43]. Cbl-b deficiency does not affect dendritic cell (DC) differentiation; however, levels of tumor necrosis factor alpha (TNF) and interleukin 6 (IL-6) were significantly increased in *Cbl-b*-/- mice after LPS-induced TLR4 stimulation [29]. The enhanced cytokine production by DCs does not result in the expansion of antigen-specific CD8+ T cells in the *Cbl-b*-/- mice. Downregulation of Cbl-b in CD4+ T cells has recently been reported in systemic lupus erythematosus (SLE) patients [45], and Cbl-b-deficient SLE patients exhibited T follicular helper (Tfh) cell hyper-responses.

Exhaustion and dysfunction of effector CD8+ T-cells in the tumor microenvironment (TME) is a major limiting factor for T cell-mediated immunity against solid tumors. T cells that undergo exhaustion progressively lose their effector functions, including the expression of interferon gamma (IFN-γ) and TNF. Instead, they express the inhibitory receptors PD-1, Tim3, and LAG3 and lose their ability to kill tumor cells [46]. The *Cbl-b* transcript was shown to be preferentially upregulated in PD-1+ Tim3+ exhausted CD8+ tumor-infiltrating lymphocytes (TILs) in multiple tumor models [31,47], as well as in a lymphocytic choriomeningitis virus (LCMV) infection model [48]. It appears, therefore, that Cbl-b supports the exhaustion state of T cells and negatively regulates their functions [49]. In agreement, Cbl-b-depleted T cells are highly effective in the rejection of transplanted, chemically induced, and spontaneously arising tumors [13,25]. A recent report demonstrated that CD8+ TILs exhibit upregulated Cbl-b, which coincides with the dysfunctional state of the TILs [31]. Deleting *Cbl-b* by CRISPR/Cas9-based genome editing restored the expression of cytokines and effector molecules, including IFN-γ, TNF, granzyme B, and IL-2, in PD-1+Tim3+ CD8+ TILs [31].

In general, *Cbl-b*-deficient mice demonstrate hyperacute responses to both infection and cancer. *Cbl-b*-/- mice infected with LCMV showed elevated levels of IFN-γ in CD8+ T cells [50]. Similarly, the majority of *Cbl-b*-/- mice were found to spontaneously reject UVB-induced tumors [25], while adoptively transferred *Cbl-b*-/- CD8+ T cells led to the eradication of TC-1-injected tumors [25]. Although Cbl-b downregulation is an effective adjunct for inactivated vaccines, its complete absence in T cells may lead to a severe cytokine storm, including mortality, as was demonstrated in certain *Cbl-b* knockout mouse models [25].

In a recently reported study, distinct homozygous mutations in CBL-B were identified in three unrelated children with early-onset autoimmune diseases. T cells in response to anti-CD3 cross-linking from these patients exhibited hyperproliferation. p.R496X mutation abolished CBL-B expression, while p.C464W mutation preserved CBL-B expression. The third mutation, p.H285L, was expressed at half the normal level in the patient’s cells. CBL-B p.H257L mutation in mice, which corresponds to p.H285L human mutation, had hyperproliferating T and B cells in response to antigen receptor cross-linking. Cbl-bH257L mice also had increased percentages of Tregs. However, T effector cells from the patient with the p.H285L mutation and Cbl-bH257L mice were resistant to suppression by WT Tregs [51].

## 4. Cbl and Chimeric Antigen Receptor (CAR) T Cells

CAR T-cell based therapy represents a major breakthrough in cancer care. One of the limitations of this method is the potential loss of expression of the single-chain variable fragment (scFv) and its poor persistence in the patients’ lymphoid cells [52]. Recent work revealed that ubiquitin-mediated endocytosis facilitated the degradation of the CAR T receptors. In this study, increased stability of CAR T was obtained by mutating the lysine residues in the cytoplasmic domain of the CAR T construct, leading to long-lasting CAR T-cell responses in mouse models [53].

As observed in the exhausted CD8+ T-cells, an upregulated Cbl-b was also found in exhausted CAR T cells within the tumor microenvironment [47]. Recently, a second-generation CAR construct against human carcinoembryonic antigen (hCEAscFV-CD28--CD3ζ) was generated to target human colorectal adenocarcinomas. A considerable survival benefit of mice implanted with MC38-CEA tumor cells was observed in Cbl-b-deficient over Cbl-b-sufficient CAR T cells [31]. Exhausted PD-1+Tim3+ T cells were markedly reduced among the TILs of mice that were adoptively transfused with *Cbl-b*–/– CAR T cells. While isolated *Cbl-b*–/–PD-1+Tim3+ CEA-CAR T cells exhibited tumor cell killing ability when challenged with MC38-CEA in vitro, the analogous *Cbl-b*+/+ cells were markedly limited in their tumor killing ability. Moreover, cultured *Cbl-b*–/– CEA-CAR T cells, but not the analogous *Cbl-b* +/+ T cells, expressed effector molecules like IFN-γ, TNF, and granzyme B.

The use of retroviral vectors in human CAR therapies is currently limited due to the concern of potential insertional mutagenesis. The actively transcribed genes are the sites of the viral vector integration, and therefore this process poses a potential risk of mutagenesis [54,55]. In a recent attempt to treat B cell lymphoma, an asymptotic rapid clonal expansion of infused CAR T cells was observed [56]. Since the CD22-CAR T cells were manufactured using the lentiviral transduction procedure, the lentiviral vector integration site was determined. Interestingly, the analysis revealed that the vector integrated into the second intron of the Cbl-b gene [56]. The single insertion of the CAR vector might have resulted in a dominant-negative effect or impairment of Cbl-b function, resulting in the clonal expansion.

## 5. Involvement of Cbl in the Regulation of T Cell Activation

Signaling from the T-cell antigen receptor is tightly regulated, and the extent of activation of T cells is dependent on the relative rates of receptor recycling to the membrane versus its internalization and proteasomal degradation. Ubiquitination of the TCR/CD3 complexes [18,19] occurs following antigen-mediated receptor stimulation, which leads to the internalization of the receptor complex [9]. Cbl-b appears to be a major regulator of this process in which it contributes to the internalization of the TCR/CD3 complex [57]. Cbl-b ubiquitinates selected proteins that are involved in the early activation events in T cells and modulates the clustering of effector molecules in a way that destabilizes the immune synapse and attenuates TCR signaling [58].

Cbl-b is a major regulator of signal transduction in T cells that exhibits inhibitory effects on CD28-mediated T cell signaling. Evidently, Cbl-b-deficient T cells can undergo activation independent of CD28-mediated signals [16], and in vivo deficiency of Cbl-b can rescue T-cell impairments observed in CD28 knockout mice [24]. The PI3K is activated downstream of the CD28-signaling pathway and is inhibited by Cbl-b-mediated ubiquitination of its p85β subunit [59] and consequential inhibition of p85β interaction with CD28 [60]. In the same line, increased Akt activation and NF-κB signaling were observed in *Cbl-b-*/*-* T cells [43]. A concomitant negative mechanism of feedback inhibition can be activated by CD28 that disables the Cbl-b-inhibitory pathways by promoting post-translational modifications of Cbl-b, leading to its proteasomal degradation [61,62]. This mechanism is initiated by CD28 signaling-mediated inhibition of the SHP1 phosphatase [63] and the Lck and PKCθ kinase-mediated phosphorylation of Cbl-b [63,64], which results in Cbl-b ubiquitination and proteasomal degradation [64]. In a recent study, we found that the intracellular adapter protein, CT-10 regulator of kinases (Crk)-II, can simultaneously interact with Cbl-b and the Crk SH3 domain-binding guanine nucleotide-releasing factor (C3G) [65]. CrkII utilizes its SH2 domain to transiently associate with Cbl-b in either resting or activated T cells and its SH3N domain to constitutively interact with C3G. The Crk adaptor proteins play key roles in signal transduction in T cells, where they link downstream effector molecules to activated receptors, thereby regulating T cell proliferation, adhesion, and migration [66]. The C3G guanine-nucleotide-exchange factor plays a role in TCR-linked signaling pathways that promote T cell adhesion [67]. Therefore, the observation that Crk proteins can form trimolecular complexes with Cbl-b and C3G [68,69] suggested a negative role for Cbl-b during T cell activation.

The association of Cbl-b and potentially other effector molecules with CrkII peaks within the first minute post TCR crosslinking and leads to the formation of multiple Cbl-b-CrkII-containing protein complexes of different molecular masses. Ubiquitination of C3G commences at ~5 min post TCR/CD3 stimulation, followed by its rapid degradation. The ubiquitination and degradation of C3G conversely correlate with the expression levels of CrkII and are dependent on the presence of the CrkII-bound Cbl-b protein [65]. It appears, therefore, that CrkII in TCR-stimulated T cells serves as a scaffold that allows Cbl-b to be in close proximity with C3G. TCR-stimulation-dependent phosphorylation of Cbl-b leads to its activation and the apparent ubiquitination of C3G. At this stage, C3G undergoes degradation and Cbl-b dissociates from CrkII, while the expression levels of Cbl-b and CrkII remain intact.

**Figure 2:**
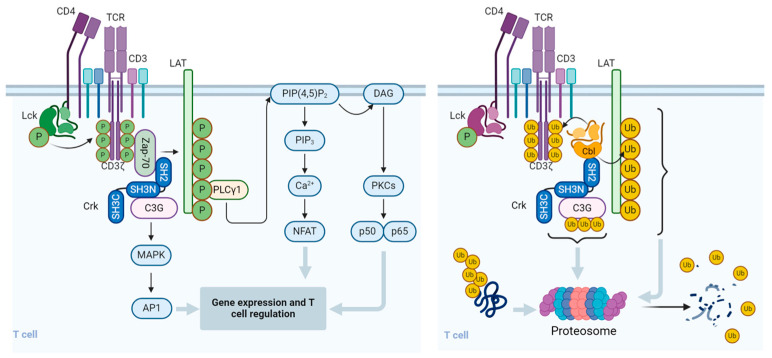
Regulation of signal transduction pathways and function of T cells by Cbl. Upon binding to the cognate peptidyl-MHC complex and stabilization by the CD4/CD8 co-receptor, the T cell receptor (TCR)/CD3 complex initiates a number of downstream activation signals that eventually determine cell fate through regulating cytokine production, cell survival, proliferation, and differentiation. An early event in TCR activation is phosphorylation of immunoreceptor tyrosine-based activation motifs (ITAMs) on the cytosolic side of the TCR/CD3 complex by lymphocyte protein tyrosine kinase (Lck). The CD45 receptor tyrosine phosphatase modulates the phosphorylation and activation of Lck that is brought by the CD4 or CD8 co-receptor to the proximity of the TCR/CD3 complex. Zeta-chain-associated protein kinase (Zap-70) is recruited to the TCR/CD3 complex, where it becomes activated, promoting recruitment and phosphorylation of downstream adaptor or scaffold proteins, like CT10 regulator of kinase (Crk). Crk recruits C3G (a guanine nucleotide exchange factor) that in turn activates the Ras/Rap1 signaling pathways, the MAPK/Erk pathways, and the AP1 transcription factor. ZAP-70 also phosphorylates the scaffold protein, Linker for activation of T cells (LAT), which recruits phospholipase C γ1 (PLCγ1), which hydrolyzes phosphatidylinositol 4,5-bisphosphate (PIP2) to form the diacylglycerol (DAG) and inositol trisphosphate (IP3) second messengers. DAG activates PKCθ and the MAPK/Erk pathways, both promoting transcription factor NF-κB. IP3 triggers the release of Ca2+ from the ER, which promotes the entry of extracellular Ca2+ into cells through calcium release-activated Ca2+ (CRAC) channels. Calcium-bound calmodulin (Ca2+/CaM) activates the phosphatase calcineurin, which promotes IL-2 gene transcription through the transcription factor NFAT. One of the effects of the Cbl-b protein in T cells is to downregulate the CD3- and CD28-linked signal transduction pathways. Cbl-b can ubiquitinate the T cell antigen receptor (TCR) CD3ζ chain and the LAT adapter protein and direct them to proteasomal degradation. In resting T cells, the CrkII adapter protein constitutively associates with the guanine nucleotide exchange factor C3G and maintains a basal level of signaling that is insufficient for cell activation. However, upon TCR stimulation, CrkII also interacts with Cbl-b (via a distinct protein–protein binding site) and brings Cbl-b into close proximity with C3G to enable the ubiquitination of C3G by Cbl-b. The ubiquitinated C3G undergoes proteasomal degradation, thereby promoting signal termination and preventing persistent immune cell activation. The figure was created using BioRender.com (www.biorender.com, accessed on 14 July 2024).

Since the presence of CrkII s essential for the ubiquitination and degradation of C3G, these results suggest that termination of TCR-stimulation-induced activation signals is regulated by CrkII-dependent Cbl-b-mediated activity. This mechanism may contribute to the fine-tuning of T cells and determine the length and intensity of the immune response (Figure 2).

## 6. Effect of Cbl on Natural Killer (NK) Cells

Being the major innate lymphoid cells (ILC), NK cells mediate cytotoxicity and regulate the immune system by secreting inflammatory cytokines such as IFN-γ and TNF [70,71]. The two major types of NK cells include the conventional NK cells (cNK), which are located in the bone marrow and peripheral blood, and the tissue-resident NK cells (trNK), which reside predominantly in the lymph nodes, thymus, lung, liver, small intestine, and uterus [72]. The differentiation as well as maturation of NK cells, in contrast to T cells, is not regulated by the Cbl-b protein. However, *Cbl-b*-/- mice display enhanced NK cell proliferation and activation [38], which is balanced by the expression of activator and inhibitory receptors that helps maintain immune surveillance and avoid the undesired autoimmune reactivity. On the contrary, activation of human NK cells results in increased expression of the Cbl-b protein [73], suggesting a negative feedback role. *Cbl-B* knockdown in resting as well as IL-15-activated NK cells resulted in enhanced cytotoxicity and expression of effector molecules, such as granzyme B, perforin and interferon gamma (IFN-γ). A direct correlation between the effects of certain cytokines and Cbl-b expression levels was reported in NK cells in which either IL-15 or IL-2 induced increased Cbl-b expression, whereas IL-7, IL-12, IL-18, and IL-21 had no effect on the expression levels of Cbl-b. Similarly, increased expression levels of Cbl-b were observed in NK cells cocultured with MHC-I-negative/NK cell-sensitive K562 cells. In contrast, NK cells showed no killing activity nor induction of Cbl-b expression when cocultured with MHC-I-sufficient/NK cell-resistant tumor cells (e.g., Molm-13 and MV4-11). NK cell-mediated lysis of Molm-13 and MV4-11 cells was observed upon siRNA-mediated Cbl-b knockout in the NK cells. Mechanistically, granzyme B and IFN-γ mRNA levels were significantly elevated in the Cbl-b-depleted NK cells. In a recent work, CRISPR/Cas9-mediated knockout of Cbl-b in human NK cells derived from placental CD34+ cells enhanced the NK cell responses against tumor cells [74]. Placental-derived NK cells were effective killers of many human tumor cell lines [75], while artificially generated Cbl-b-deficient NK cells from placental stem cells augmented the NK cell cytotoxicity toward myeloma and leukemia target cells [74].

Analysis of signal transduction pathways in NK cells suggests that Cbl-b operates downstream of the TAM tyrosine receptor kinases (TYRO3, AXL, and MER). The TAM receptors are negative regulators of NK cell activation [76]. They undergo Cbl-b-mediated ubiquitination upon interaction with their cognitive ligand, which promotes their internalization [38]. TAM activation by their cognitive ligand, growth arrest-specific gene-6 (GAS6), leads to suppression of WT NK cell expansion but not the expansion and cytokine production of Cbl-b−/− NK cells. While Cbl-b depletion of NK cells promoted metastatic mouse melanoma growth, a TAM tyrosine kinase receptor small molecule inhibitor, LDC1267, was shown to reverse this outcome [77]. Phosphorylation of human NK cell-specific TAM receptor MerTK was significantly increased in Cbl-b-depleted NK cells. MerTK inhibitors downregulated STAT5 phosphorylation in NK cells cultured with IL-15 [73], suggesting that Cbl-b also regulates MerTK phosphorylation, thereby limiting the activation signals in NK cells.

**Figure 3:**
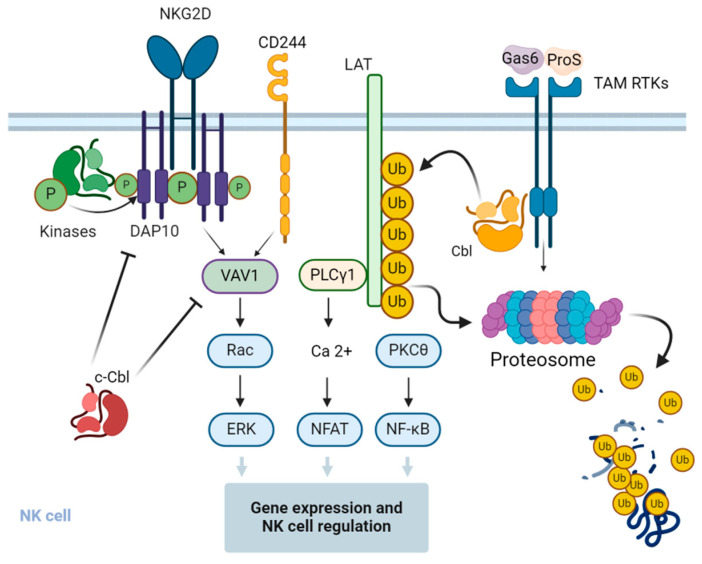
Regulation of signal transduction pathways and function of NK cells by Cbl. c-Cbl ubiquitinates the stimulatory receptor NKG2D on NK cells, leading to the endocytosis and degradation of the activation signaling complex. c-Cbl also mediates non-degradative ubiquitination of VAV1, a regulator of signal transduction pathways downstream of the NKG2D and 2B4 stimulatory receptors, and negatively regulates their downstream signaling pathways in NK cells. Upon interaction with the cognitive ligands, the growth arrest-specific gene 6 (Gas6) and protein S (ProS) and the Tyro3, Axl, and Mer (TAM)-family receptor tyrosine kinases (RTKs) phosphorylate and activate Cbl-b, which in turn promotes the ubiquitination of the adapter protein linker for activation of T cells (LAT), followed by its proteasomal degradation. This results in the inhibition of LAT-dependent signaling pathways that otherwise promote cytokine and chemokine production in NK cells. The figure was created using BioRender.com (www.biorender.com, accessed on 14 July 2024).

A recent study suggested a feedback loop mechanism where Cbl-b operates downstream of the TAM-signaling pathway. Upon interaction with their cognate ligands, TAM receptors in NK cells are shown to promote tyrosine phosphorylation and activation of Cbl-b, which mediates ubiquitination and degradation of key activation signaling adapter protein LAT1. As a result, NK cell activation is downregulated due to a block of signaling pathways upstream for the induction and production of various cytokines and chemokines [76] (Figure 3). These findings suggest that the TAM/Cbl-b inhibitory pathway may be a potential target to unleash the effector functions of NK cells.

## 7. Cbl and NK Cell Exhaustion

*Cbl-b*−/− as well as *Cbl-b C373A* (a ligase-defective Cbl) mouse NK cells demonstrated enhanced cytokine production and tumor toxicity, indicating the important role of the ubiquitin ligase activity of Cbl-b in the regulation of NK cell function [78]. This study brings new insights into the critical role of Cbl-b in negatively targeting tumor metastasis through the ubiquitination of TAM receptors. Based on this model, a proposal of a TAM/Cbl-b inhibitory pathway causing NK cell exhaustion and dysfunction has evolved. The characterization of the NK cell exhaustion mechanisms and the attempts to restore their immune activity are the main focus of multiple investigations [79]. While T cell exhaustion has been extensively studied using viral infection models, NK cell exhaustion has been primarily focused on identifying dysfunctional tumor-associated NK cells [80,81,82,83]. Reduction in the expression of IFN-γ, granzyme B, TNF, CD107a (also termed LAMP-1, a marker of degranulation in NK and CD8+ T cells), CD16-dependent antibody-dependent cell-mediated cytotoxicity (ADCC), or reduced tumor cell killing, are the hallmarks of “dysfunctional” NK cells. NK cell “exhaustion” is also associated with a concomitant increase in markers like PD-1, TIGIT, TIM-3, LAG-3, etc., which has been observed in cancer patients and tumor microenvironment-derived NK cells [80,81,82,83,84,85,86,87]. Similar phenotypic changes in NK cells are also observed in the context of chronic viral infections and prolonged exposure to pro-inflammatory cytokines [88,89,90,91]. It has been found that Cbl-b promotes NK cell dysfunction by ubiquitinating the intracellular signaling protein caspase-recruitment domain (CARD) membrane-associated guanylate kinase (MAGUK) protein 1 (CARMA1) [77], which in turn blocks NF-kB transcriptional activity, leading to suppression of NK cell function.

## 8. Cbl Inhibitors and Applications in Immunotherapy

Immune checkpoint blockade revolutionized the field of cancer immunotherapy, yet the majority of the patients develop resistance. Therefore, efforts have been made to bypass the resistance by targeting intracellular mediators. The E3 ubiquitin ligase Cbl-b is a potential target that is regulated by both CD28 and CTLA4 signaling pathways. While signaling through CD28 inhibits Cbl-b-mediated immunosuppression, CTLA-4 ligation promotes Cbl-b activity [92]. Due to its unfavorable chemical structure, Cbl-b has long been known as an ‘undruggable’ target [25,93,94]. The advent of high-throughput small-molecule and in silico screening approaches has made it possible to develop novel Cbl-b inhibitors that have reversed immunosuppression of the TME and promoted tumor-specific T cell activation and tumor regression.

T cell receptor-engineered T cells (TCR T cells) are alternatives to CAR T cells for tumor immunotherapy. A small molecule inhibitor targeting Cbl-b (Cbl-b-IN-1) prior to T cell activation promoted TCR expression efficiency, T cell proliferation, and cell survival in both PBMCs and TCR T cells. Cbl-b-IN-1-treated T cells maintained a less differentiated state with enhanced cytokine production while effectively enhancing the activation of TCR signaling, as evidenced by increased phosphorylation levels of Zeta-chain-associated protein kinase 70 (ZAP70) and phospholipase c-γ1 (PLCγ1) [95].

Nurix, a USA-based biotech company, has utilized a computational approach using a DNA-encoded library screening platform to identify compounds that link Cbl-b with proteins of interest [96]. NX-1607, identified by this method, can target signaling proteins for ligase-mediated ubiquitination and proteasomal degradation. Oral administration of NX-1607 has significantly reduced tumor growth in murine models of colon and breast cancer [97]. In these models, NX-1607 enhanced the activity of tumor-infiltrating NK cells and CD8+ T cells. Upon depletion of NK and CD8+ T cells, the drug was no longer effective in regressing the tumor, indicating its direct effect in activating cytotoxic immune cells [97]. NX-1607 is under investigation in a phase 1 clinical trial (NCT05107674).

The reduction of TCR diversity and tumor-reactive TILs is evident following checkpoint therapy. With the advancement of the drug-enhanced tumor-infiltrating lymphocyte (DeTIL) therapy platform, Nurix has developed the ex vivo Cbl-b inhibitor NX-0255 to be administered with adoptive T cell therapy. Tumor fragments derived from patients are cultured with IL-2 and NX-0255 to further enhance the cytotoxic response of TILs, which are then reinfused back in patients. NX-0255 is also under clinical investigation for advanced tumors and recruitment has started for a phase 1 trial (NCT05107739).

HotSpot Therapeutics has developed a small molecule Cbl-b inhibitor (HOT-A) using allosteric chemistry [98]. HOT-A prevents Cbl-b phosphorylation and functional activity by blocking a novel regulatory pocket on Cbl-b [99]. This molecule increases IFNγ secretion and CD8+ T cell proliferation in human PBMCs as well as enhanced cytotoxicity of NK cells. HOT-A-injected mice showed enhanced IL-2 secretion and increased differentiation of activated (CD3+/CD69+) T cells [99]. HOT-A is also under clinical investigation for advanced tumors and started recruitment for a phase 1 trial (NCT05662397).

APN401 is a siRNA-mediated Cbl-b knockdown approach developed by Apeiron. This approach utilizes the electroporation method to transfect Cbl-b-specific siRNA into T cells and combines it with adoptive T cell therapy. siRNA-transfected CD8+ T cells together with a DC-based tumor vaccine showed enhanced intratumoral inflammatory cytokine signaling and an augmented B16 melanoma inhibition in a murine model [39]. However, preliminary data from a phase I clinical trial showed the best tumor response occurred in only four of sixteen patients [100]. The lack of immune-related adverse events (irAE) with this platform is encouraging and leaves room for further improvement of its translational application.

## 9. Recently Discovered Mechanism of Cbl Regulation in Activated Immune Cells

T cell and NK cell hyperproliferation and exacerbated inflammatory responses can be detrimental to the host and are therefore normally tuned down by an array of regulatory mechanisms. Protein ubiquitination is among the most effective post-translational mechanisms that regulate the proteins’ fate and determine their structure, function, and quantity. This process is mediated by E3 ubiquitin ligases that catalyze the ubiquitination of specific lysine residues and promote the proteasomal degradation of the targeted molecules.

The Cbl-b E3 ubiquitin ligase is an abundant protein in lymphoid cells, including T cells and NK cells. It recruits to the immunological synapse of TCR-stimulated T cells at the T cell-contact area with the antigen-presenting cells (APC) [14]. Cbl-b interacts with the TCRζ chain and with a number of TCR downstream effector molecules, including Lck, ZAP70, Vav, and PKCθ [16,21,101,102]. Moreover, by forming a trimolecular complex with Crk and C3G, it delivers signals that downregulate T cell activation [16,69,103]. Utilizing transient expression of CrkII mutants in Jurkat T cells in co-immunoprecipitation studies, we found that the formation of Cbl-b-CrkII complexes is T cell activation-dependent and mediated by binding of the Cbl-b phosphotyrosine epitopes to the CrkII SH2 domain. On the other hand, the association of the C3G protein with CrkII is cell activation-independent and is mediated by C3G interaction with the CrkII-SH3N domain. The rate of C3G ubiquitination is significantly increased in T cells that overexpress CrkII, while downregulation of CrkII coincides with a reduction in C3G ubiquitination. Furthermore, increased ubiquitination of C3G directly correlated with the reduction in its expression level, suggesting that C3G ubiquitination provides a signal for its degradation. In contrast to C3G, CrkII and Cbl-b protein expression levels were not altered during the first half an hour post-TCR stimulation. Nevertheless, Cbl-b, which physically interacts with CrkII in TCR/CD3-stimulated T cells, dissociates from CrkII at a much faster rate in CrkII-overexpressing T cells, apparently because a large fraction of the C3G proteins in the CrkII–Cbl-b–C3G complexes undergoes ubiquitination and degradation. The formation of Cbl-b- and C3G-containing protein complexes in activated T cells requires the presence of CrkII since such complexes can be formed in CrkII-sufficient but not in CrkII-deficient T cells. The recent findings imply that CrkII in TCR/CD3-activated T cells functions as a scaffold for Cbl-b and C3G, whereby the close proximity of the two proteins enables the ubiquitination of C3G by Cbl-b [65].

The potential mechanism by which the CrkII–Cbl-b–C3G-containing trimolecular complexes contribute to the signaling pathways downstream of the activated TCR/CD3 receptors appears to be very complicated due to the fact that T cells express three different isoforms of Crk, including CrkI, CrkII, and CrkL, and three distinct isoforms of CBL-B. Two of the Cbl isoforms, c-Cbl and Cbl-b, exhibit a high degree of structural and functional homology and appear to ubiquitinate several different effector proteins that participate in signal transduction from activated TCR [104,105].

The CrkL isoform can also associate with Cbl-b [20] and C3G [106] and form CrkL–Cbl-b–C3G trimolecular complexes [69,107]. CrkL is a substrate for Cbl-b and can undergo ubiquitination, predominantly in TCR/CD3-stimulated T cells. However, this ubiquitination does not promote the degradation of Crk-L, rather reducing its binding affinity to C3G.

Crk-L association with C3G increases following knockdown of Cbl-b, resulting in enhanced Rap1 and LFA-1 activity. These results support a model in which Cbl-b functions as a negative regulator of the CrkL–C3G signaling pathway in TCR/CD3-stimulated T cells [69]. The current findings substantiate the hypothesis that both CrkII and CrkL function as adaptors for Cbl-b and C3G and contribute to the negative regulation and termination of the TCR/CD3-linked activation signals. The overall effects of CrkII and CrkL in activated T cells appear to be similar, although these two adaptor proteins impose their effects by utilizing two different biochemical mechanisms.

## 10. Conclusions and Future Directions

Ubiquitination is an essential biological process carried out by multistep reaction cascades that control protein homeostasis and regulate signal transduction from a wide range of cell surface receptors. It is essential for the functional regulation of all types of immunocytes, including T cells and NK cells. Data obtained from experimental models, in addition to a large number of preclinical studies, support the idea that targeting ubiquitination and related factors is effective in improving cancer immunotherapies. Over forty different clinical trials targeting Cbl-b in a wide range of ailments, including various types of leukemia and lymphoma, adenocarcinoma, lung cancer, uveitis, and other diseases, are either in progress or have been completed in the past two decades (Supplementary Table S1), implying the potential impact of Cbl-b in this field.

As mammalian cells express a large number of E3s and DUBs, utilizing ubiquitin-dependent therapeutic approaches has a huge prospect in cancer drug development. Therefore, characterizing and understanding further the functions of the novel E3s and DUBs in antitumor immune responses should be the focus of future studies. The development of small-molecule inhibitors will promote more preclinical and clinical studies and enrich the drug inventory for cancer immunotherapy. More effort should also be spent on preclinical studies, including the improvement of adoptive cell therapy and the procedures for the specific knock-out or knock-down of selected E3s and DUBs. These approaches will be of particular relevance for CAR-based adoptive T cell and NK cell therapies.

## Supplementary Materials

The following supporting information can be downloaded at: https://www.mdpi.com/article/10.3390/life14121592/s1, Table S1. List of clinical trials targeting Cbl.

## Abbreviations

ADCC	antibody-dependent cell-mediated cytotoxicity
APC	antigen-presenting cells
C3G	Crk SH3 domain-binding guanine nucleotide-releasing factor
CAR	chimeric antigen receptor
CARD	caspase recruitment domain
CARMA1	CARD-MAGUK protein 1
Cbl	Casitas B-lineage lymphoma
cNK	conventional NK cells
CRK	CT-10 regulator of kinases
DC	dendritic cell
DUB	deubiquitinase
GAS6	growth arrest-specific gene-6
IL	interleukin
ILC	innate lymphoid cells
IFN-γ	interferon gamma
LAT	linker of activation of T cells
Lck	cell-specific protein-tyrosine kinase
LCMV	lymphocytic choriomeningitis virus
LZ	leucine zipper
MAGUK	membrane-associated guanylate kinase
NK	natural killer
PI3K	phosphoinositide-3 kinase
PKCθ	protein kinase C theta
PLCγ1	phospholipase C gamma 1
PR	proline rich
RING	really interesting group finger domain
SLE	systemic lupus erythematosus
SLP76	SH2-domain-containing leukocyte protein of 76 kDa
TAM	Tyro3, Axl, and Mer (receptor kinases)
TCR	T cell antigen receptor
Tfh	follicular helper T cell
TGF-β	transforming growth factor beta
TILs	tumor-infiltrating lymphocytes
TKB	tyrosine kinase binding
TNFα	tumor necrosis factor alpha
trNK	tissue-resident NK cells
UB	ubiquitin
ZAP70	zeta chain-associated protein 70

## References

[1] Kuwabara T., Matsui Y., Ishikawa F., Kondo M. (2018). Regulation of T-Cell Signaling by Post-Translational Modifications in Autoimmune Disease. Int. J. Mol. Sci..

[2] Raposo B., Merky P., Lundqvist C., Yamada H., Urbonaviciute V., Niaudet C., Viljanen J., Kihlberg J., Kyewski B., Ekwall O. (2018). T cells specific for post-translational modifications escape intrathymic tolerance induction. Nat. Commun..

[3] Sun S.-C. (2008). Deubiquitylation and regulation of the immune response. Nat. Rev. Immunol..

[4] Deng L., Meng T., Chen L., Wei W., Wang P. (2020). The role of ubiquitination in tumorigenesis and targeted drug discovery. Signal Transduct. Target. Ther..

[5] Li J., Chai Q.Y., Liu C.H. (2016). The ubiquitin system: A critical regulator of innate immunity and pathogen–host interactions. Cell. Mol. Immunol..

[6] Malynn B.A., Ma A. (2010). Ubiquitin Makes Its Mark on Immune Regulation. Immunity.

[7] Hu H., Sun S.-C. (2016). Ubiquitin signaling in immune responses. Cell Res..

[8] Pickart C.M. (2001). Mechanisms Underlying Ubiquitination. Annu. Rev. Biochem..

[9] Haglund K., Dikic I. (2012). The role of ubiquitylation in receptor endocytosis and endosomal sorting. J. Cell Sci..

[10] Hicke L. (2001). Protein regulation by monoubiquitin. Nat. Rev. Mol. Cell Biol..

[11] Tsygankov A.Y., Teckchandani A.M., Feshchenko E.A., Swaminathan G. (2001). Beyond the RING: CBL proteins as multivalent adapters. Oncogene.

[12] Meng W., Sawasdikosol S., Burakoff S.J., Eck M.J. (1999). Structure of the amino-terminal domain of Cbl complexed to its binding site on ZAP-70 kinase. Nature.

[13] Chiang J.Y., Jang I.K., Hodes R., Gu H. (2007). Ablation of Cbl-b provides protection against transplanted and spontaneous tumors. J. Clin. Investig..

[14] Wiedemann A., Müller S., Favier B., Penna D., Guiraud M., Delmas C., Champagne E., Valitutti S. (2005). T-cell activation is accompanied by an ubiquitination process occurring at the immunological synapse. Immunol. Lett..

[15] Garcia G.G., Miller R.A. (2001). Single-Cell Analyses Reveal Two Defects in Peptide-Specific Activation of Naive T Cells from Aged Mice. J. Immunol..

[16] Bachmaier K., Krawczyk C., Kozieradzki I., Kong Y.Y., Sasaki T., Oliveira-Dos-Santos A., Mariathasan S., Bouchard D., Wakeham A., Itie A. (2000). Negative regulation of lymphocyte activation and autoimmunity by the molecular adaptor Cbl-b. Nature.

[17] Dou H., Buetow L., Hock A., Sibbet G.J., Vousden K.H., Huang D.T. (2012). Structural basis for autoinhibition and phosphorylation-dependent activation of c-Cbl. Nat. Struct. Mol. Biol..

[18] Hou D., Cenciarelli C., Jensen J.P., Nguygen H.B., Weissman A.M. (1994). Activation-dependent ubiquitination of a T cell antigen receptor subunit on multiple intracellular lysines. J. Biol. Chem..

[19] Cenciarelli C., Hou D., Hsu K.C., Rellahan B.L., Wiest D.L., Smith H.T., Fried V.A., Weissman A.M. (1992). Activation-Induced Ubiquitination of the T Cell Antigen Receptor. Science.

[20] Andoniou C.E., Lill N.L., Thien C.B., Lupher M.L., Ota S., Bowtell D.D.L., Scaife R.M., Langdon W.Y., Band H. (2000). The Cbl Proto-Oncogene Product Negatively Regulates the Src-Family Tyrosine Kinase Fyn by Enhancing Its Degradation. Mol. Cell. Biol..

[21] Rao N., Miyake S., Reddi A.L., Douillard P., Ghosh A.K., Dodge I.L., Zhou P., Fernandes N.D., Band H. (2002). Negative regulation of Lck by Cbl ubiquitin ligase. Proc. Natl. Acad. Sci. USA.

[22] Huang H., Jeon M.S., Liao L., Yang C., Elly C., Yates J.R., Liu Y.-C. (2010). K33-Linked Polyubiquitination of T Cell Receptor-ζ Regulates Proteolysis-Independent T Cell Signaling. Immunity.

[23] Zhang R., Zhang N., Mueller D.L. (2008). Casitas B-Lineage Lymphoma b Inhibits Antigen Recognition and Slows Cell Cycle Progression at Late Times during CD4 ^+^ T Cell Clonal Expansion. J. Immunol..

[24] Chiang Y.J., Kole H.K., Brown K., Naramura M., Fukuhara S., Hu R.J., Jang I.K., Gutkind J.S., Shevach E., Gu H. (2000). Cbl-b regulates the CD28 dependence of T-cell activation. Nature.

[25] Loeser S., Loser K., Bijker M.S., Rangachari M., Van Der Burg S.H., Wada T., Beissert S., Melief C.J.M., Penninger J.M. (2007). Spontaneous tumor rejection by *cbl-b*–deficient CD8^+^ T cells. J. Exp. Med..

[26] Singh T.P., Vieyra-Garcia P.A., Wagner K., Penninger J., Wolf P. (2018). Cbl-b deficiency provides protection against UVB-induced skin damage by modulating inflammatory gene signature. Cell Death Dis..

[27] Stromnes I.M., Blattman J.N., Tan X., Jeevanjee S., Gu H., Greenberg P.D. (2010). Abrogating Cbl-b in effector CD8+ T cells improves the efficacy of adoptive therapy of leukemia in mice. J. Clin. Investig..

[28] Paolino M., Thien C.B.F., Gruber T., Hinterleitner R., Baier G., Langdon W.Y., Penninger J.M. (2011). Essential Role of E3 Ubiquitin Ligase Activity in Cbl-b–Regulated T Cell Functions. J. Immunol..

[29] Lutz-Nicoladoni C., Wolf D., Sopper S. (2015). Modulation of Immune Cell Functions by the E3 Ligase Cbl-b. Front Oncol..

[30] Schanz O., Cornez I., Yajnanarayana S.P., David F.S., Peer S., Gruber T., Krawitz P., Brossart P., Heine A., Landsberg J. (2021). Tumor rejection in Cblb^−/−^ mice depends on IL-9 and Th9 cells. J. Immunother. Cancer.

[31] Kumar J., Kumar R., Singh A.K., Tsakem E.L., Kathania M., Riese M.J., Theiss A.L., Davila M.L., Venuprasad K. (2021). Deletion of Cbl-b inhibits CD8^+^ T-cell exhaustion and promotes CAR T-cell function. J. Immunother. Cancer.

[32] Nguyen T.T.T., Wang Z.E., Shen L., Schroeder A., Eckalbar W., Weiss A. (2021). Cbl-b deficiency prevents functional but not phenotypic T cell anergy. J. Exp. Med..

[33] Jeon M.S., Atfield A., Venuprasad K., Krawczyk C., Sarao R., Elly C., Yang C., Arya S., Bachmaier K., Su L. (2004). Essential Role of the E3 Ubiquitin Ligase Cbl-b in T Cell Anergy Induction. Immunity.

[34] Wohlfert E.A., Callahan M.K., Clark R.B. (2004). Resistance to CD4^+^CD25^+^ Regulatory T Cells and TGF-β in Cbl-b^−/−^ Mice. J. Immunol..

[35] Fujiwara M., Anstadt E.J., Clark R.B. (2017). Cbl-b Deficiency Mediates Resistance to Programed Death-Ligand 1/Programed Death-1 Regulation. Front. Immunol..

[36] Peer S., Baier G., Gruber T. (2017). *Cblb*-deficient T cells are less susceptible to PD-L1-mediated inhibition. Oncotarget.

[37] Gronski M.A., Boulter J.M., Moskophidis D., Nguyen L.T., Holmberg K., Elford A.R., Deenick E.K., Kim H.O., Penninger J.M., Odermatt B. (2004). TCR affinity and negative regulation limit autoimmunity. Nat. Med..

[38] Paolino M., Choidas A., Wallner S., Pranjic B., Uribesalgo I., Loeser S., Jamieson A.M., Langdon W.Y., Ikeda F., Fededa J.P. (2014). The E3 ligase Cbl-b and TAM receptors regulate cancer metastasis via natural killer cells. Nature.

[39] Hinterleitner R., Gruber T., Pfeifhofer-Obermair C., Lutz-Nicoladoni C., Tzankov A., Schuster M., Penninger J.M., Loibner H., Lametschwandtner G., Wolf D. (2012). Adoptive Transfer of siRNA Cblb-Silenced CD8+ T Lymphocytes Augments Tumor Vaccine Efficacy in a B16 Melanoma Model. PLoS ONE.

[40] Thell K., Urban M., Harrauer J., Haslinger I., Kuttke M., Brunner J.S., Vogel A., Schabbauer G., Penninger J., Gaweco A. (2019). Master checkpoint Cbl-b inhibition: Anti-tumour efficacy in a murine colorectal cancer model following siRNA-based cell therapy. Ann. Oncol..

[41] Triozzi P., Kooshki M., Alistar A., Bitting R., Neal A., Lametschwandtner G., Loibner H. (2015). Phase I clinical trial of adoptive cellular immunotherapy with APN401 in patients with solid tumors. J. Immunother. Cancer.

[42] Qiao G., Ying H., Zhao Y., Liang Y., Guo H., Shen H., Li Z., Solway J., Tao E., Chiang Y.J. (2014). E3 Ubiquitin Ligase Cbl-b Suppresses Proallergic T Cell Development and Allergic Airway Inflammation. Cell Rep..

[43] Qiao G., Zhao Y., Li Z., Tang P.Q., Langdon W.Y., Yang T., Zhang J. (2013). T Cell Activation Threshold Regulated by E3 Ubiquitin Ligase Cbl-b Determines Fate of Inducible Regulatory T Cells. J. Immunol..

[44] Han S., Chung D.C., St Paul M., Liu Z.Q., Garcia-Batres C., Elford A.R., Tran C.W., Chapatte L., Ohashi P.S. (2020). Overproduction of IL-2 by Cbl-b deficient CD4(+) T cells provides resistance against regulatory T cells. OncoImmunology.

[45] Li X., Sun W., Huang M., Gong L., Zhang X., Zhong L., Calderon V., Bian Z., He Y., Suh W.-K. (2024). Deficiency of CBL and CBLB ubiquitin ligases leads to hyper T follicular helper cell responses and lupus by reducing BCL6 degradation. Immunity.

[46] McLane L.M., Abdel-Hakeem M.S., Wherry E.J. (2019). CD8 T Cell Exhaustion During Chronic Viral Infection and Cancer. Annu. Rev. Immunol..

[47] Chen J., López-Moyado I.F., Seo H., Lio C.W.J., Hempleman L.J., Sekiya T., Yoshimura A., Scott-Browne J.P., Rao A. (2019). NR4A transcription factors limit CAR T cell function in solid tumours. Nature.

[48] Scott-Browne J.P., López-Moyado I.F., Trifari S., Wong V., Chavez L., Rao A., Pereira R.M. (2016). Dynamic Changes in Chromatin Accessibility Occur in CD8 + T Cells Responding to Viral Infection. Immunity.

[49] Venuprasad K. (2010). Cbl-b and Itch: Key Regulators of Peripheral T-cell Tolerance. Cancer Res..

[50] Shamim M., Nanjappa S.G., Singh A., Plisch E.H., LeBlanc S.E., Walent J., Svaren J., Seroogy C., Suresh M. (2007). Cbl-b Regulates Antigen-Induced TCR Down-Regulation and IFN-γ Production by Effector CD8 T Cells without Affecting Functional Avidity. J. Immunol..

[51] Janssen E., Peters Z., Alosaimi M.F., Smith E., Milin E., Stafstrom K., Wallace J.G., Platt C.D., Chou J., El Ansari Y.S. (2022). Immune dysregulation caused by homozygous mutations in *CBLB*. J. Clin. Investig..

[52] Gauthier J., Yakoub-Agha I. (2017). Chimeric antigen-receptor T-cell therapy for hematological malignancies and solid tumors: Clinical data to date, current limitations and perspectives. Curr. Res. Transl. Med..

[53] Li W., Qiu S., Chen J., Jiang S., Chen W., Jiang J., Wang F., Si W., Shu Y., Wei P. (2020). Chimeric Antigen Receptor Designed to Prevent Ubiquitination and Downregulation Showed Durable Antitumor Efficacy. Immunity.

[54] Wu X., Li Y., Crise B., Burgess S.M. (2003). Transcription Start Regions in the Human Genome Are Favored Targets for MLV Integration. Science.

[55] Schröder A.R.W., Shinn P., Chen H., Berry C., Ecker J.R., Bushman F. (2002). HIV-1 Integration in the Human Genome Favors Active Genes and Local Hotspots. Cell.

[56] Shah N.N., Qin H., Yates B., Su L., Shalabi H., Raffeld M., Ahlman M.A., Stetler-Stevenson M., Yuan C., Guo S. (2019). Clonal expansion of CAR T cells harboring lentivector integration in the CBL gene following anti-CD22 CAR T-cell therapy. Blood Adv..

[57] Naramura M., Jang I.K., Kole H., Huang F., Haines D., Gu H. (2002). c-Cbl and Cbl-b regulate T cell responsiveness by promoting ligand-induced TCR down-modulation. Nat. Immunol..

[58] Krawczyk C., Bachmaier K., Sasaki T., Jones R.G., Snapper S.B., Bouchard D., Kozieradzki I., Ohashi P.S., Alt F.W., Penninger J.M. (2000). Cbl-b Is a Negative Regulator of Receptor Clustering and Raft Aggregation in T Cells. Immunity.

[59] Alcázar I., Cortés I., Zaballos A., Hernandez C., Fruman D.A., Barber D.F., Carrera A.C. (2009). p85β phosphoinositide 3-kinase regulates CD28 coreceptor function. Blood.

[60] Fang D., Liu Y.-C. (2001). Proteolysis-independent regulation of PI3K by Cbl-b–mediated ubiquitination in T cells. Nat. Immunol..

[61] Li D., Gál I., Vermes C., Alegre M.-L., Chong A.S.F., Chen L., Shao Q., Adarichev V., Xu X., Koreny T. (2004). Cutting Edge: Cbl-b: One of the Key Molecules Tuning CD28- and CTLA-4-Mediated T Cell Costimulation. J. Immunol..

[62] Zhang J., Bárdos T., Li D., Gál I., Vermes C., Xu J., Mikecz K., Finnegan A., Lipkowitz S., Glant T.T. (2002). Cutting Edge: Regulation of T Cell Activation Threshold by CD28 Costimulation Through Targeting Cbl-b for Ubiquitination. J. Immunol..

[63] Xiao Y., Qiao G., Tang J., Tang R., Guo H., Warwar S., Langdon W.Y., Tao L., Zhang J. (2015). Protein Tyrosine Phosphatase SHP-1 Modulates T Cell Responses by Controlling Cbl-b Degradation. J. Immunol..

[64] Gruber T., Hermann-Kleiter N., Hinterleitner R., Fresser F., Schneider R., Gastl G., Penninger J.M., Baier G. (2009). PKC-θ Modulates the Strength of T Cell Responses by Targeting Cbl-b for Ubiquitination and Degradation. Sci. Signal.

[65] Nath P.R., Anto N.P., Braiman A., Isakov N. (2023). Termination of TCR-mediated activation signals is regulated by CrkII-dependent Cbl-mediated ubiquitination and degradation of C3G. Immunobiology.

[66] Nath P.R., Dong G., Braiman A., Isakov N. (2014). Immunophilins Control T Lymphocyte Adhesion and Migration by Regulating CrkII Binding to C3G. J. Immunol..

[67] Nolz J.C., Nacusi L.P., Segovis C.M., Medeiros R.B., Mitchell J.S., Shimizu Y., Billadeau D.D. (2008). The WAVE2 complex regulates T cell receptor signaling to integrins via Abl- and CrkL–C3G-mediated activation of Rap1. J. Cell Biol..

[68] Reedquist K.A., Fukazawa T., Panchamoorthy G., Langdon W.Y., Shoelson S.E., Druker B.J., Band H. (1996). Stimulation through the T Cell Receptor Induces Cbl Association with Crk Proteins and the Guanine Nucleotide Exchange Protein C3G. J. Biol. Chem..

[69] Zhang W., Shao Y., Fang D., Huang J., Jeon M.S., Liu Y.C. (2003). Negative Regulation of T Cell Antigen Receptor-mediated Crk-L-C3G Signaling and Cell Adhesion by Cbl-b. J. Biol. Chem..

[70] Nath P.R., Gangaplara A., Pal-Nath D., Mandal A., Maric D., Sipes J.M., Cam M., Shevach E.M., Roberts D.D. (2018). CD47 Expression in Natural Killer Cells Regulates Homeostasis and Modulates Immune Response to Lymphocytic Choriomeningitis Virus. Front. Immunol..

[71] Nath P.R., Pal-Nath D., Mandal A., Cam M.C., Schwartz A.L., Roberts D.D. (2019). Natural Killer Cell Recruitment and Activation Are Regulated by CD47 Expression in the Tumor Microenvironment. Cancer Immunol. Res..

[72] Nath P.R., Maclean M., Nagarajan V., Lee J.W., Yakin M., Kumar A., Nadali H., Schmidt B., Kaya K.D., Kodati S. (2024). Single-cell profiling identifies a CD8bright CD244bright Natural Killer cell subset that reflects disease activity in HLA-A29-positive birdshot chorioretinopathy. Nat. Commun..

[73] Lu T., Chen L., Mansour A.G., Yu M.J., Brooks N., Teng K.-Y., Li Z., Zhang J., Barr T., Yu J. (2021). Cbl-b Is Upregulated and Plays a Negative Role in Activated Human NK Cells. J. Immunol..

[74] Guo X., Mahlakõiv T., Ye Q., Somanchi S., He S., Rana H., DiFiglia A., Gleason J., van der Touw W., Hariri R. (2021). CBLB ablation with CRISPR/Cas9 enhances cytotoxicity of human placental stem cell-derived NK cells for cancer immunotherapy. J. Immunother. Cancer.

[75] Kang L., Voskinarian-Berse V., Law E., Reddin T., Bhatia M., Hariri A., Ning Y., Dong D., Maguire T., Yarmush M. (2013). Characterization and ex vivo Expansion of Human Placenta-Derived Natural Killer Cells for Cancer Immunotherapy. Front. Immunol..

[76] Chirino L.M., Kumar S., Okumura M., Sterner D.E., Mattern M., Butt T.R., Kambayashi T. (2020). TAM receptors attenuate murine NK-cell responses via E3 ubiquitin ligase Cbl-b. Eur. J. Immunol..

[77] Kojo S., Elly C., Harada Y., Langdon W.Y., Kronenberg M., Liu Y.-C. (2009). Mechanisms of NKT cell anergy induction involve Cbl-b-promoted monoubiquitination of CARMA1. Proc. Natl. Acad. Sci. USA.

[78] Augustin R.C., Bao R., Luke J.J. (2023). Targeting Cbl-b in cancer immunotherapy. J. Immunother. Cancer.

[79] Judge S.J., Murphy W.J., Canter R.J. (2020). Characterizing the Dysfunctional NK Cell: Assessing the Clinical Relevance of Exhaustion, Anergy, and Senescence. Front. Cell. Infect. Microbiol..

[80] Da Silva I.P., Gallois A., Jimenez-Baranda S., Khan S., Anderson A.C., Kuchroo V.K., Osman I., Bhardwaj N. (2014). Reversal of NK-Cell Exhaustion in Advanced Melanoma by Tim-3 Blockade. Cancer Immunol. Res..

[81] Beldi-Ferchiou A., Lambert M., Dogniaux S., Vély F., Vivier E., Olive D., Dupuy S., Levasseur F., Zucman D., Lebbé C. (2016). PD-1 mediates functional exhaustion of activated NK cells in patients with Kaposi sarcoma. Oncotarget.

[82] Seo H., Jeon I., Kim B.S., Park M., Bae E.A., Song B., Koh C.-H., Shin K.-S., Kim I.-K., Choi K. (2017). IL-21-mediated reversal of NK cell exhaustion facilitates anti-tumour immunity in MHC class I-deficient tumours. Nat. Commun..

[83] Zhang Q., Bi J., Zheng X., Chen Y., Wang H., Wu W., Wang Z., Wu Q., Peng H., Wei H. (2018). Blockade of the checkpoint receptor TIGIT prevents NK cell exhaustion and elicits potent anti-tumor immunity. Nat. Immunol..

[84] Benson D.M., Bakan C.E., Mishra A., Hofmeister C.C., Efebera Y., Becknell B., Baiocchi R.A., Zhang J., Yu J., Smith M.K. (2010). The PD-1/PD-L1 axis modulates the natural killer cell versus multiple myeloma effect: A therapeutic target for CT-011, a novel monoclonal anti–PD-1 antibody. Blood.

[85] MacFarlane A.W., Jillab M., Plimack E.R., Hudes G.R., Uzzo R.G., Litwin S., Dulaimi E., Al-Saleem T., Campbell K.S. (2014). PD-1 Expression on Peripheral Blood Cells Increases with Stage in Renal Cell Carcinoma Patients and Is Rapidly Reduced after Surgical Tumor Resection. Cancer Immunol. Res..

[86] Vari F., Arpon D., Keane C., Hertzberg M.S., Talaulikar D., Jain S., Cui Q., Han E., Tobin J., Bird R. (2018). Immune evasion via PD-1/PD-L1 on NK cells and monocyte/macrophages is more prominent in Hodgkin lymphoma than DLBCL. Blood.

[87] Sun H., Huang Q., Huang M., Wen H., Lin R., Zheng M., Qu K., Li K., Wei H., Xiao W. (2019). Human CD96 Correlates to Natural Killer Cell Exhaustion and Predicts the Prognosis of Human Hepatocellular Carcinoma. Hepatology.

[88] Wiesmayr S., Webber S.A., Macedo C., Popescu I., Smith L., Luce J., Metes D. (2012). Decreased NKp46 and NKG2D and elevated PD-1 are associated with altered NK-cell function in pediatric transplant patients with PTLD. Eur. J. Immunol..

[89] Felices M., Lenvik A.J., McElmurry R., Chu S., Hinderlie P., Bendzick L., Geller M.A., Tolar J., Blazar B.R., Miller J.S. (2018). Continuous treatment with IL-15 exhausts human NK cells via a metabolic defect. JCI Insight.

[90] Alvarez M., Simonetta F., Baker J., Pierini A., Wenokur A.S., Morrison A.R., Murphy W.J., Negrin R.S. (2019). Regulation of murine NK cell exhaustion through the activation of the DNA damage repair pathway. JCI Insight.

[91] Zhang C., Wang X.M., Li S.R., Twelkmeyer T., Wang W.H., Zhang S.Y., Wang S.-F., Chen J.-Z., Jin X., Wu Y.-Z. (2019). NKG2A is a NK cell exhaustion checkpoint for HCV persistence. Nat. Commun..

[92] Chen W., Jin W., Wahl S.M. (1998). Engagement of cytotoxic T lymphocyte-associated antigen 4 (CTLA-4) induces transforming growth factor beta (TGF-beta) production by murine CD4(+) T cells. J. Exp. Med..

[93] Tang R., Langdon W.Y., Zhang J. (2019). Regulation of immune responses by E3 ubiquitin ligase Cbl-b. Cell. Immunol..

[94] Dang C.V., Reddy E.P., Shokat K.M., Soucek L. (2017). Drugging the ‘undruggable’ cancer targets. Nat. Rev. Cancer.

[95] Wang J., Han X., Hao Y., Chen S., Pang B., Zou L., Han X., Wang W., Liu L., Shen M. (2024). Cbl-b inhibition promotes less differentiated phenotypes of T cells with enhanced cytokine production. Cell. Immunol..

[96] Clark M.A., Acharya R.A., Arico-Muendel C.C., Belyanskaya S.L., Benjamin D.R., Carlson N.R., Centrella P.A., Chiu C.H., Creaser S.P., Cuozzo J.W. (2009). Design, synthesis and selection of DNA-encoded small-molecule libraries. Nat. Chem. Biol..

[97] Rountree R., Cohen F., Tenn-McClellan A., Borodovsky A., Gallotta M., Stokes J., Romo J.G., Karim C., Hansen G.M., Guiducci C. (2021). Abstract 1595: Small molecule inhibition of the ubiquitin ligase CBL-B results in potent T and NK cell mediated anti-tumor response. Cancer Res..

[98] Sheik Amamuddy O., Veldman W., Manyumwa C., Khairallah A., Agajanian S., Oluyemi O., Verkhivker G.M., Tastan Bishop Ö. (2020). Integrated Computational Approaches and Tools forAllosteric Drug Discovery. Int. J. Mol. Sci..

[99] Kuai J., Bi Y., Qi Y., Conrady D., Govindaraj R., Hone G., Denny R.A., Carson K., Harriman G., Wang F. (2021). 864 Identification of a novel allosteric oral Cbl-b inhibitor that augmented T cell response and enhanced NK cell killing in vitro and in vivo. J. Immunother. Cancer.

[100] Loibner H., Lametschwandtner G., Westritschnig K., Mutschlechner O., Dohnal A., Salzberg M.O., Triozzi P.L. (2018). Adoptive cellular immunotherapy with APN401, autologous cbl-b silenced peripheral blood mononuclear cells: Data from a phase I study in patients with solid tumors. J. Clin. Oncol..

[101] Wang H.Y., Altman Y., Fang D., Elly C., Dai Y., Shao Y., Liu Y.-C. (2001). Cbl Promotes Ubiquitination of the T Cell Receptor ζ through an Adaptor Function of Zap-70. J. Biol. Chem..

[102] Lupher M.L., Reedquist K.A., Miyake S., Langdon W.Y., Band H. (1996). A Novel Phosphotyrosine-binding Domain in the N-terminal Transforming Region of Cbl Interacts Directly and Selectively with ZAP-70 in T Cells. J. Biol. Chem..

[103] Thien C.B.F., Langdon W.Y. (2005). c-Cbl and Cbl-b ubiquitin ligases: Substrate diversity and the negative regulation of signalling responses. Biochem. J..

[104] Liu Y.C., Gu H. (2002). Cbl and Cbl-b in T-cell regulation. Trends Immunol..

[105] Liu Y.C. (2004). Ubiquitin Ligases and the Immune Response. Annu. Rev. Immunol..

[106] Smit L., Van Der Horst G., Borst J. (1996). Sos, Vav, and C3G Participate in B Cell Receptor-induced Signaling Pathways and Differentially Associate with Shc-Grb2, Crk, and Crk-L Adaptors. J. Biol. Chem..

[107] Uemura N., Griffin J.D. (1999). The Adapter Protein Crkl Links Cbl to C3G after Integrin Ligation and Enhances Cell Migration. J. Biol. Chem..

